# A Low Daily Intake of Simple Sugars in the Diet Is Associated with Improved Liver Function in Cirrhotic Liver Transplant Candidates

**DOI:** 10.3390/nu15071575

**Published:** 2023-03-24

**Authors:** Simona Parisse, Sara Carnevale, Francesca Di Bartolomeo, Edoardo Poli, Francesca Miceli, Flaminia Ferri, Monica Mischitelli, Bianca Rocco, Quirino Lai, Pierleone Lucatelli, Mario Corona, Gianluca Mennini, Adriano De Santis, Massimo Rossi, Maurizio Muscaritoli, Alfredo Cantafora, Stefano Ginanni Corradini

**Affiliations:** 1 Department of Translational and Precision Medicine, Sapienza University of Rome, 00185 Rome, Italy; 2 Belcolle Hospital, 01100 Viterbo, Italy; 3 Centre Hépato-Biliaire, Hôpital Paul Brousse, AP-HP, 94800 Villejuif, France; 4 Department of Radiological, Oncological and Anatomopathological Sciences, Sapienza University of Rome, 00161 Rome, Italy; 5 General Surgery and Organ Transplantation Unit, Department of Surgery, Sapienza University of Rome, 00161 Rome, Italy

**Keywords:** cirrhosis, simple sugars, liver transplant, DELTA-MELD, cirrhotic diet, visceral adipose tissue

## Abstract

(1) Background: We investigated, for the first time, whether dietary simple sugar intake affects MELD score changes over time in a cohort of cirrhotic liver transplant candidates. (2) Methods: the MELD score, dietary habits using a 3-day food diary, and visceral adipose tissue index (VATI) measured with CT scan were assessed in 80 consecutive outpatient cirrhotic patients at baseline, after counseling to follow current nutritional guidelines. The MELD score was reassessed after six months and the DELTA-MELD was calculated as the MELD at the second assessment minus the MELD at baseline. (3) Results: Compared with the baseline, the MELD score of cirrhotic patients at the end of the study was decreased, stable, or increased in 36%, 8% and 56% of patients, respectively. In separate multiple linear regression models, DELTA-MELD was positively and independently correlated with the daily intake of simple sugars expressed in g/kg body weight (*p* = 0.01) or as a percentage of total caloric intake (*p* = 0.0004) and with the number of daily portions of fruit, added sugar, jam, and honey (*p* = 0.003). These associations were present almost exclusively in patients with VATI above the median value. (4) Conclusions: In cirrhotic patients with high amounts of visceral adipose tissue the consumption of simple sugars and fructose should be limited to improve their clinical outcome.

## 1. Introduction

Cirrhosis is a chronic liver disease, often associated with poor outcomes. The total number of patients diagnosed with liver cirrhosis worldwide in 2019 was 1602.43 million, equivalent to a global prevalence of 20.7% [1]. The number of cases of cirrhosis in 1990 was 1075.12 million, and then, in the following 29 years, it increased by 49% [1]. Cirrhosis was the 11th leading cause of death worldwide in 2019 [2]. The age-standardized global mortality rate of cirrhosis of the liver was 16.66 per 100,000 in 1990 and 17.31 per 100,000 in 2017, representing an increase of 47.2% in 27 years [3].

The MELD score and its changes over time are predictive parameters for survival of cirrhotic patients both in general and for patients on the liver transplant (LT) waiting list [4,5]. LT represents the only curative treatment for patients with liver cirrhosis. Some LT candidates during their prelisting assessment or when already on the waiting list may experience clinical improvement with a reduction in the MELD score. If this improvement significantly reduces the MELD score, in the absence of hepatocellular carcinoma (HCC) or other conditions that impair quality of life, their prognosis without transplant becomes better than without surgery and they are not placed on the waiting list or are delisted [6]. Furthermore, an inadequate nutritional status, both undernutrition and overnutrition, represents a factor associated with increased morbidity, clinical decompensation, worsening of the MELD score, and increased mortality in cirrhotic patients in general and in LT candidates [7].

Regarding daily dietary habits and their clinical implications in cirrhosis, there is a growing awareness of the role of total calorie intake, which differs according to the patient’s nutritional status, and of some nutrients (e.g., protein, sodium, and branched-chain amino acids) in the evolution of this disease and in the onset of clinically relevant complications [8,9,10]. Recently, since the documented clinical relevance of nutrition in the evolution of cirrhosis, international scientific societies have oriented their efforts toward the identification of common dietary and lifestyle recommendations to ameliorate the nutritional status and dietary habits of patients affected by chronic liver diseases and cirrhosis [7,11,12,13]. However, considering the guidelines of international scientific societies and also the recommendations recently published outside them, the consumption of oral carbohydrates by cirrhotic patients is little addressed, with only a few exceptions and some conflicting points that can be summarized as follows: (a) carbohydrates should account for different percentages of caloric intake ranging from 50–60% to 45–75% [13,14,15]; (b) in overweight/obese patients, a reduction in the intake of carbohydrates and fat is recommended as an indirect consequence of the recommendation to follow a low-calorie and high-protein diet [7,11,13,16,17]; (c) the possibility or the recommendation of using carbohydrates, according to some authors preferably complex carbohydrates, in evening snacks that play a beneficial role in limiting the duration of fasting periods [11,12,13,16]; (d) the recommendation of eating plenty of fruits and vegetables contained in the EASL guidelines [7]; (e) in contrast with the latter recommendation, preferring complex carbohydrates and not allowing simple sugars to exceed 10–15% of the total caloric intake [13,15]. This last recommendation, which is not contained in the recent nutritional guidelines of international scientific societies for cirrhotic patients, is derived from the guidelines of the World Health Organization on sugar consumption in the general population, which recommend a consumption lower than 10% of total caloric intake [18].

Indeed, the daily consumption of simple sugars has never been investigated in cirrhotic patients in general nor in those candidates for LT in relation to clinical outcomes. This represents an important knowledge gap for two reasons. First, because patients with cirrhosis related to non-alcoholic fatty liver disease (NAFLD) or metabolic associated fatty liver disease (MAFLD) are steadily increasing overall and among LT candidates [19,20,21,22,23,24]. Secondly, because it is increasingly evident that in patients with NAFLD or MAFLD in the noncirrhotic phase, hepatic fat accumulation, inflammation, and hepatic fibrosis are associated with the consumption of simple sugars, in particular fructose and sucrose [25,26,27,28,29].

Therefore, the present study aims to investigate, in a cohort of outpatient cirrhotic patients visited at the Liver Transplant Center of the La Sapienza University of Rome, the daily dietary consumption of monosaccharides and disaccharides and its association with changes over time of the MELD score.

## 2. Materials and Methods

We prospectively enrolled 111 consecutive patients with cirrhosis, liver transplant candidates, visited for the first time at the LT Outpatient Service of the Gastroenterology Division of the Sapienza University of Rome. The inclusion criteria were: age above 18 years; diagnosis of liver cirrhosis made by clinical, laboratory, and imaging techniques; stable clinical conditions; and obtaining informed consent. The main exclusion criteria were age older than 70 years, active or recent (less than 6 months) alcohol consumption, evidence of an ongoing acute decompensation of cirrhosis, diagnosis of heart failure (NYHA ≥ II class) and respiratory failure (GOLD III and IV), hepatocellular carcinoma (HCC) exceeding Milan criteria for LT or other extrahepatic malignancies, and previous LT. Two control groups for dietary habits were identified, the first of 15 healthy subjects and the second of 25 patients with chronic viral hepatitis.

All subjects were instructed by a trained dietitian at enrollment to complete a 3-day food diary over the next 10 days. Demographic, nutritional, and clinical data were collected for each subject at the time of enrollment.

In cirrhotic patients the MELD score was calculated not only at enrollment but also after 6 months for the DELTA-MELD calculation. In order to adjust for baseline visceral adiposity, the impact of simple sugar consumption on DELTA-MELD, the visceral adipose tissue index (VATI), was measured within 30 days of enrollment using computed tomography.

**Figure 1:**
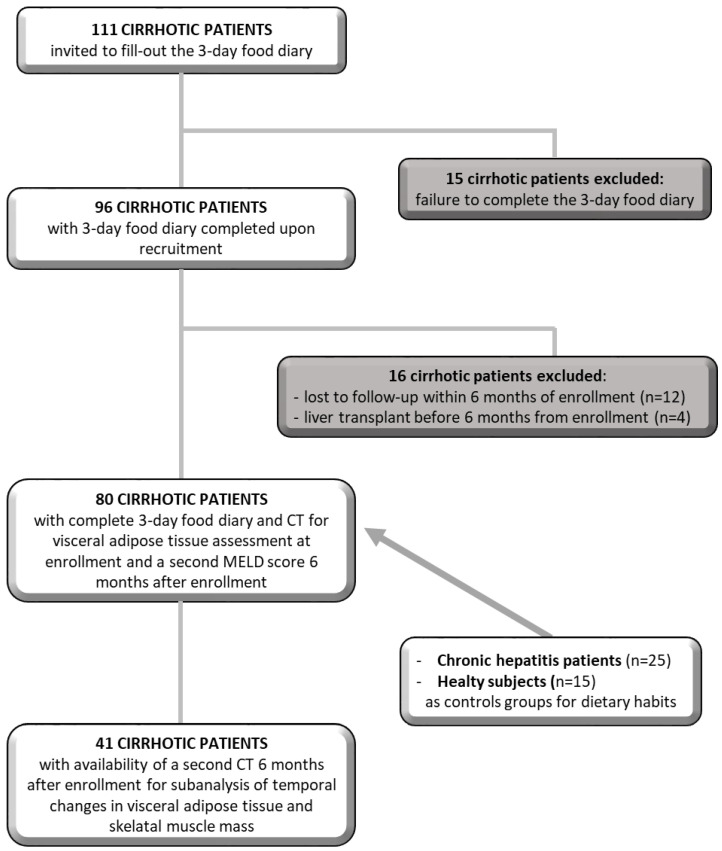
Schematic diagram depicting the study population.

Among the 111 cirrhotic patients enrolled, only 96 patients completed the 3-day food diary and another 16 patients were excluded because they were lost to follow-up (*n* = 12) or because they underwent LT (*n* = 4) within 6 months of registration. Therefore, the study includes 80 cirrhotic patients. To verify possible associations between the consumption of soluble sugars and changes in visceral fat and muscle mass, we used a second CT that a subgroup of 41 cirrhotic patients had done for clinical reasons after 6 months from enrollment. We then compared the VATI and skeletal muscle index (SMI) measured at enrollment with those after 6 months (Figure 1). The study was performed in accordance with the Declaration of Helsinki, approved by the Ethics Committee of Policlinico Umberto I (Ref. No. 3420), and written informed consent was obtained from all participants.

### 2.1. Anthropometric and Nutritional Assessment

The body mass index (BMI) was calculated as body weight in kg divided by the square of the height in meters. For those patients with ascites and/or edema, the body weight was estimated with one of the following methods: (a) calculating dry body weight by subtracting a percentage from the measured weight based on ultrasound evaluation of ascites and clinical evaluation of edema, (b) using the weight after evacuation paracentesis, or (c) using known weight before fluid retention. Subjective global nutritional assessment (SGA) was performed for every enrolled patient. The SGA was obtained considering the personal history of nutrient intake and weight loss, the presence of gastrointestinal symptoms, and patient functional capacity, together with physical signs of malnutrition (for example, depletion of subcutaneous fat and muscle mass) [30]. Patients were classified as well nourished (SGA A), moderately malnourished (SGA B), or severely malnourished (SGA C). To minimize inter-operator differences, these assessments were carried out by the same clinician.

For every patient, the same expert clinician carried out the measurements of mid-arm circumference (MAC) and of triceps skinfold thickness. MAC was evaluated with a flexible tape measure at halfway between the acromion and olecranon process of the nondominant arm, with both arms hanging at the sides. Measurements of the triceps skinfold thickness were obtained using a Harpender skinfold caliper (John Bull British Indicators Ltd., St Albans, UK), following recommended protocols [31]. All measurements were taken from the body’s left side to the nearest 0.1 mm at the midpoint between the acromion and the olecranon process by exerting a pressure of 10 g/mm^3^.

### 2.2. Dietary Habits

The total caloric intake and daily dietary nutrient consumption were prospectively collected using a 3-day food diary realized over two nonconsecutive weekdays and a weekend day in the week following enrollment [32]. None of the participants had food allergies and/or food intolerances. Upon enrollment, each subject was provided by an expert dietitian with detailed instructions for completing the diary. After compilation, an expert dietitian revised the records through a face-to-face interview employing a food atlas to obtain accurate and complete records [33]. The energy and nutrient composition of the foods were derived from the Food Composition Database for Epidemiological Studies in Italy (Banca Dati di Composizione degli Alimenti per Studi Epidemiologici in Italia—BDA) (European Institute of Oncology, 2015) [34,35]. The nutritional data was counted as the average daily intake of the three days covered by the diary. The total energy intake was expressed in Kcal per day (Kcal/day), while the daily dietary intake of the macronutrient categories under study was expressed as grams per kilogram of body weight (g/Kg) or as a percentage of total caloric intake (%). The carbohydrate categories extrapolated from the BDA database were: (1) total carbohydrates; (2) starches; and (3) simple sugars, i.e., the sum of glucose, fructose, galactose, sucrose, maltose, and lactose. Particular attention was then paid to the types of foods that contain simple sugars, such as: (a) fruit; (b) added sugars, jams, and honey; (c) sugary sweets; (d) vegetables; (e) milk and yogurt; (f) soft drinks; and (g) fruit juices. The daily portions of each of these categories were calculated from the 3-day food diary in compliance with the 2014 indications of the Italian Society of Human Nutrition (SINU) on standard portions for the Italian population [36]. All patients with cirrhosis were asked not to change their dietary habits during the study period. Furthermore, no dietary interventions were made other than the current nutritional recommendations for cirrhosis [7].

### 2.3. DELTA-MELD Computation

Liver disease severity was classified according to the MELD score derived from the laboratory data measured at enrollment using standard laboratory methods. To determine possible temporal changes in liver disease severity, the MELD score was also recorded 6 months after enrollment and its changes over time were expressed as DELTA-MELD, counted by the following formula: [(MELD 6 months after enrollment—MELD upon enrollment)/time expressed in days] × 180.

### 2.4. Visceral Adipose Tissue and Skeletal Muscle Mass Evaluation

The radiological measurements of nutritional status parameters were realized employing abdominal images from CT scans taken at the third lumbar disc plane (L3) level, realized during the local standard LT evaluation protocol within 30 days of enrollment. Visceral adipose tissue and skeletal muscle estimation were realized using a specific software (Slice-O-Matic software, V4.2; Tomovision, Montreal, QC, Canada). Standard Hounsfield Units (HU) with thresholds of −150 to −50 HU and −30 to +150 HU were applied for the visceral adipose tissue and muscle area, respectively. Then, using these HUs, a trained operator skilled in anatomy demarked visceral adipose tissue and muscles (i.e., psoas, paraspinals, rectus abdominis, transversus abdominis, quadratus lumborum, and external and internal oblique muscles) within the abdominal wall for respective tissue cross-sectional area estimation. For each of the two tissues, the given tissue pixels were summed, multiplied by the pixel surface areas and, consequently, tissue cross-sectional areas (cm^2^) were obtained. VATI and SMI were computed by dividing each of the two tissue cross-sectional areas by the square of the patient height expressed in meters.

### 2.5. Statistical Analysis

The continuous variables were not normally distributed in the Kolmogorov–Smirnov test, and data were expressed as the median and interquartile range. For categorical variables, data were expressed as absolute values and percentages. To test the overall differences among groups, continuous variables were analyzed with the Mann–Whitney test and, for categorical variables, using χ^2^.

To verify whether, in patients with cirrhosis, the intake of various types of carbohydrates in the diet was associated with changes in the MELD score 6 months after enrollment with respect to the baseline value, we first performed univariate analyzes by dividing the patients into two groups: (a) those that showed improvement (negative values of DELTA-MELD) and (b) those who remained stable or showed a worsening prognosis (DELTA-MELD values ≥ 0). Subsequently we wanted to verify if there were linear correlations independent of confounders between the intake of the various types of carbohydrates and the change over time of the MELD score. We built several multivariate linear regression models by inserting the DELTA-MELD as the dependent variable in each of them. Only one of the dietary factors was introduced into each model as an independent variable along with the confounders. Model selection was performed with the backward stepwise and bootstrap elimination method. Tests were two-tailed, and a *p*-value < 0.05 was considered statistically significant. Analysis was performed using SPSS software 25.0 for Windows (SPSS Inc., Chicago, IL, USA).

## 3. Results

### 3.1. Dietary Nutrient Intake in Cirrhotic Patients Compared with That in Chronic Hepatitis Patients and Healthy Controls

Table 1 shows the demographic characteristics, clinical variables of the metabolic syndrome, and nutritional features of patients with liver cirrhosis, those with chronic hepatitis, and healthy controls. There were no intergroup differences in age. Cirrhotic patients were more frequently male than subjects with chronic hepatitis. BMI and the frequency of overweight/obesity were higher in cirrhotic patients than in the other two groups. The frequency of diabetes, dyslipidemia, and arterial hypertension was 26%, 16%, and 24%, respectively, in cirrhotic patients, but equal to zero in the other two groups. Regarding nutritional assessment, cirrhotic patients appeared globally well nourished, with a median dry body weight of 80.1 (IQR 68.0–93.2) Kg. Only six cirrhotic patients were classified as SGA B and one as SGA C. No intergroup differences were present for the MAC values and triceps skinfold measurements. Regarding daily food consumption, we found that compared with healthy controls, cirrhotic patients ate significantly fewer total calories, proteins, fats, and servings of sugary sweets, but more servings of vegetables and fruits. Furthermore, compared with patients with chronic hepatitis, cirrhotic patients consumed significantly fewer servings of milk and yogurt, but more of vegetables. We found no intergroup differences in the consumption of total carbohydrates, starches, or simple sugars, or the number of added sugar, jam, and honey servings. Most subjects did not consume any servings of soft drinks; the number of subjects who consumed at least one portion over the three days of food recordings was equal to 24 (30%), 1 (4%), and 5 (32%) in cirrhotic patients, patients with chronic hepatitis, and healthy controls, respectively. Most subjects did not consume any servings of fruit juice; the number of subjects who consumed at least one portion was equal to 22 (27%), 1 (4%), and 6 (40%) in cirrhotic patients, patients with chronic hepatitis, and healthy controls, respectively. The percentages of cirrhotic patients who met the nutritional guidelines for patients with this disease regarding daily consumption of total calories and protein were 6% and 2.7%, respectively. The percentage of subjects in each group consuming less than 10% of total calories as simple sugars was 8.8%, 8.0%, and 20.0% in cirrhotic patients, patients with chronic hepatitis, and healthy controls, respectively.

### 3.2. A Low Daily Intake of Simple Sugars in the Diet Is Associated with the Improvement of the MELD Score of Cirrhotic Patients over Time

Among 80 cirrhotic patients, the MELD score measured after 6 months, compared with that measured at enrollment, was higher, equal, and lower in 45 (56%), 6 (8%), and 29 (36%) patients, respectively. Of the patients who had worsened at the second MELD score, 23 had increased MELD by at least two points. Of the patients who had improved, 8 had decreased MELD by at least two points.

We therefore wanted to verify whether, in cirrhotic patients, the change in the MELD score during a 6-month period was associated with dietary carbohydrate intake. To do this we divided the cirrhotic patients into two subgroups: one with stable or worsening disease (i.e., the MELD score was unchanged or increased and therefore DELTA-MELD ≥ 0; *n* = 51) during the 6 months of observation, and the other that had improved (i.e., the MELD score was decreased and thus DELTA-MELD < 0; *n* = 29).

As shown in Table 2, despite equal MELD values at enrollment in the two groups, after 6 months, in the group with stable or worsening disease the MELD score was significantly higher than that of the group that had clinically improved. As expected, the DELTA-MELD value was significantly lower in the improved group than in the other group. Furthermore, no statistically significant differences were found in the clinical, nutritional, and demographic characteristics between the patients of the two groups.

**Figure 2:**
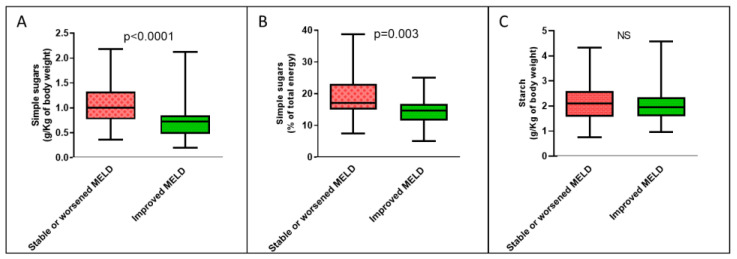
Boxplots of the amount of dietary carbohydrates consumed each day according to temporal changes in MELD score by all cirrhotic patients. (**A**) Intake of simple sugars expressed in g/Kg of body weight; (**B**) intake of simple sugars expressed as a percentage of total energy; (**C**) intake of starch. NS: not significant.

Regarding food consumption, however, we found that patients with MELD scores that improved over time showed lower median daily dietary intake of simple sugars, both expressed in grams per unit of body weight [0.73 (IQR 0.48–0.85) vs. 1.00 (IQR 0.77–1.33) g/Kg, *p* < 0.0001] and as a percentage of total caloric intake [14.6 (IQR 11.4–16.7) vs. 17.0 (IQR 15.0–23.1) %, *p* = 0.003] (Figure 2). Starch intake was equal in the two groups, while there was a nonsignificant trend for lower total carbohydrate intake in the clinical improvement group. We therefore sought to understand which food categories containing simple sugars were associated with the improvement in the MELD score over time when consumed in limited quantities.

Table 3 illustrates the absolute, not normalized for body weight, median number of servings of major foods containing simple sugars consumed in one day by cirrhotic patients with improved MELD scores and by those with stable or worsening disease. Consumption of vegetables, milk, and yogurt did not differ in the two groups. However, patients with MELD improvement compared with the other group had significantly lower daily portions of sugary sweets and the sum of added sugars, jam, and honey, while there was a nonsignificant trend for lower fruit consumption. Noteworthy, the group of patients who did not improve their MELD score consumed nearly three servings per day when adding sugar, jam, honey, and fruit combined, and nearly four servings when adding also sugary sweets. These amounts were, respectively, 1.8–1.6 times higher than those consumed by patients who improved.

The same differences between groups were present when the number of daily servings was expressed per kg of body weight (Supplementary Figure S1). Patients with MELD improvement compared with the other group had a significantly lower number of daily servings, expressed per unit of body weight, of the sum of added sugar, jam, and honey [0.000 (IQR 0.000–0.012) vs. 0.012 (IQR 0.000–0.023) servings/Kg, *p* < 0.0001] and sugary sweets [0.004 (IQR 0.000–0.008) vs. 0.010 (IQR 0.004–0.013) servings/Kg, *p* = 0.003], while there was a nonsignificant trend for a lower fruit consumption in the improved group [0.018 (IQR 0.009–0.024) vs. 0.019 (IQR 0.013–0.030) servings/Kg, *p* = 0.198].

Patients with MELD improvement compared with the other group had a significantly lower median value of the sum of the daily portions of added sugars, jam, honey, and fruit expressed per unit of body weight [0.023 (IQR 0.010–0.035) vs. 0.036 (IQR 0.023–0.050) portions/Kg, *p* = 0.001]. When sugary sweets were also added, the difference between the groups was even greater (*p* < 0.0001), being the respective median values 0.027 (IQR 0.017–0.042) and 0.048 (IQR 0.033–0.060) servings/Kg. Consumption of soft drinks, fruit juices, vegetables, milk, and yogurt was not different between cirrhotic patients who improved and those with stable or worsening disease over time. We therefore wanted to verify if, within the entire population of cirrhotic patients under study, there were any independent correlations between the changes in the MELD score over time and the food consumption mentioned above. We built separate multivariate linear regression models for each of the dietary factors, inserting DELTA-MELD as the dependent variable in all of them. As reported in Table 4, the daily dietary intake of simple sugars, expressed both in grams per unit of body weight and as a percentage of total caloric intake, were positively associated with DELTA-MELD after adjustment for confounding factors. Regarding the number of daily servings expressed per unit of body weight, fruit; the sum of added sugars, jam, and honey; and the sum of these four foods were positively associated with DELTA-MELD. Conversely, consumption of sugary sweets was not correlated with changes in MELD.

### 3.3. The Beneficial Effect of a Simple-Sugars-Restricted Diet on the MELD Score of Cirrhotic Patients Is More Pronounced in Patients with High Visceral Adipose Tissue

We then evaluated whether the association between dietary simple sugars intake and temporal changes in the MELD score of cirrhotic patients differed according to visceral adiposity. We thus subgrouped our cirrhotic study population according to median VATI values (20.6, IQR 16.3–26.7; cm^2^/m^2^). We found that cirrhotic patients with low VATI, compared with those with high VATI, did not differ in terms of DELTA-MELD [0.360 (IQR −0.392–2.890) vs. 0.159 (IQR −0.600–2.048), *p* = 0.516]. In the low-VATI group and in the high-VATI group, 14 (35%) and 15 (38%) patients showed an improvement in the MELD score at follow-up, respectively. Cirrhotic patients with low VATI, compared with those with high VATI, did not differ in terms of dietary simple sugars intake, expressed both in grams per unit of body weight [0.81 (IQR 0.70–1.15) vs. 0.86 (IQR 0.64–1.20) g/Kg, *p* = 0.765] and as a percentage of total caloric intake [15.8 (IQR 13.3–21.0) vs. 16.5 (IQR 13.7–21.6) %, *p* = 0.273].

**Figure 3:**
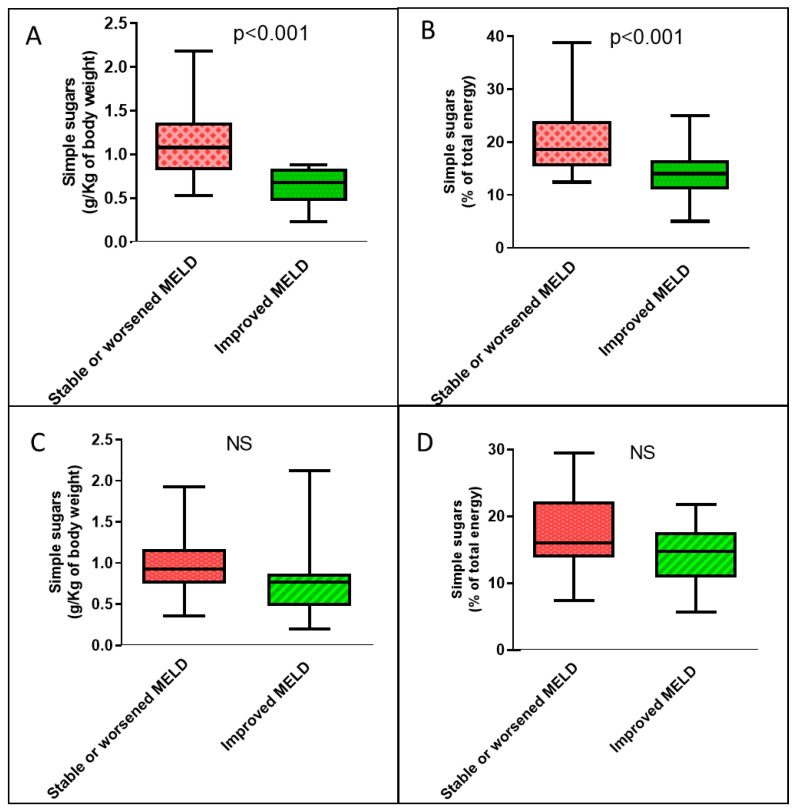
Boxplots of the amounts of simple sugars consumed each day according to temporal changes in the MELD score by cirrhotic patients according to visceral adipose tissue. (**A**) Dietary simple sugars intake expressed as g/Kg of body weight among patients with high VATI. (**B**) Dietary simple sugars intake expressed as a percentage of total energy among patients with high VATI. (**C**) Dietary simple sugars intake expressed as g/Kg of body weight among patients with low VATI. (**D**) Dietary simple sugars intake expressed as a percentage of total energy among patients with low VATI. NS: not significant.

In the group of cirrhotic patients with high VATI, the only significant difference in the demographic, clinical, and nutritional variables between the group that had an improved MELD score over time compared with that without improvement was lower VATI (Supplementary Table S1). In the low-VATI group of cirrhotic patients, there were no clinical, nutritional, or demographic differences between patients who improved their MELD score over time and patients who did not (Supplementary Table S1). With regard to dietary habits, only in the group of cirrhotic patients with high VATI was the median consumption of simple sugars, expressed both per unit of body weight and as a percentage of total calories, significantly lower in patients who subsequently experienced an improvement in MELD scores compared with those who did not improve (Figure 3).

The correlations between DELTA-MELD and food intake variables were present almost exclusively in the group of cirrhotic patients with high VATI. Indeed, in that group, DELTA-MELD was significantly associated in the multiple linear regression analyses with the daily consumption of simple sugars, expressed both in grams per unit of body weight (B = 2.285 95% CI 0.617–3.954; *p* = 0.009) and as a percentage of total caloric intake (B = 0.169 95% CI 0.055–0.283; *p* = 0.005). Furthermore, the DELTA-MELD was also associated with the number of summed daily servings of added sugar, jam, and honey, expressed per unit of body weight (B = 64.741 95% CI 3.302–126.179; *p* = 0.039) and with the sum of the servings of these three foods with fruit servings (B = 48.970 95% CI 10.436–87.503; *p* = 0.014) (Supplementary Table S2). Conversely, in the group of cirrhotic patients with low VATI there was only an association between DELTA-MELD and consumption of simple sugars expressed as a percentage of total caloric consumption (B = 0.161 95% CI 0.020–0.302; *p* = 0.026), weaker compared with that found in the group of cirrhotic patients with high VATI.

### 3.4. A Low Daily Intake of Simple Sugars in the Diet Is Associated with a Progressive Increase in the Ratio of Visceral Fat to Skeletal Muscle Mass in Cirrhotic Patients

In a subgroup of 41 cirrhotic patients, 22 with high VATI and 19 with low VATI on CT scan performed at enrollment, a second CT scan was available after a median time interval of 194 (IQR 143–334) days. At the second CT examination, compared with the first, we found the following changes: (a) 15 patients showed a decrease in VATI with a median value of −10.4 (IQR −23.8–−5.5)% and 26 patients showed an increase with a median value of 15.8 (IQR 6.5–37.2)%; (b) 27 patients showed a decrease in the SMI with a median value of −5.8 (IQR −10.7–−1.4)% and 14 patients showed an increase with a median value of 9.8 (IQR 4.1–15.9) %; (c) 15 patients showed a decrease in the VATI/SMI ratio with a median value of −14.3 (IQR −21.1–−3.8)% and 26 patients showed an increase with a median value of 23.8 (IQR 9.1–39.2)%. The median consumption of soluble sugars, expressed in grams per unit of body weight, showed a nonsignificant trend for higher values in cirrhotic patients who, over time, had increased the VATI compared with those who had decreased it (Supplementary Figure S2). Median consumption of soluble sugars was significantly lower in cirrhotic patients who, over time, increased compared with those who decreased their SMI [0.82 (IQR 0.63–1.07) vs. 0.93 (IQR 0.77–0.1.21) g/Kg; *p* = 0.043]. Median consumption of soluble sugars was significantly lower in cirrhotic patients who, over time, decreased versus increased their VATI/SMI ratio [0.77 (IQR 0.60–0.93) vs. 1.00 (IQR 0.79–1.19) g/Kg; *p* = 0.015].

## 4. Discussion

In the present study, we focused on the dietary carbohydrate consumption of cirrhotic patients being evaluated for LT. We wanted to relate carbohydrate intake to an important clinical outcome such as medium-term temporal changes in the MELD score, the most widely used disease prognostic index. The main result of our study is that the daily dietary consumption of simple sugars, but not that of starch, is positively associated with increasing values of the DELTA-MELD; i.e., with the subsequent increase over time in the MELD score. In our study, we did not recommend any changes in dietary carbohydrate intake during the follow-up, so it is likely that the consumption of simple sugars did not change substantially from that recorded at enrollment. The patients who after six months from enrollment showed negative values of the DELTA-MELD, i.e., a reduction in the score and, therefore, an improvement in the prognosis, consumed fewer simple sugars than those who did not improve.

In our study we also sought to understand which types of food containing simple sugars were most associated with changes in the MELD score over time. Our results show that the improvement over time in MELD was linearly correlated with a low consumption of added sugar, jam, and honey and, more weakly, with that of fruit. These results indirectly suggest that cirrhotic patients can improve their prognosis if they consume low amounts of fructose for the following reasons. First, glucose contained in added sugar, jam, honey, and fruit is unlikely to be involved in the association with temporal changes in MELD. This is because the MELD changes were not associated with the consumption of other glucose-rich foods that do not contain fructose, such as starch and milk, or that can contain small amounts of fructose, such as yogurt. Second, changes in MELD over time were associated with the consumption of fruit, but not with the consumption of vegetables, which contain relatively more glucose than fructose compared with fruit. In our study, the association between improvement in the MELD score over time and low fruit consumption was less strong than low consumption of added sugars, jam, and honey. This could be because fruits contain other beneficial substances such as micronutrients, phytochemicals, and fiber. The latter, in addition to playing a beneficial metabolic role, could reduce the postprandial blood concentration of fructose, in analogy with what happens to glucose when it is ingested in foods containing fiber [37].

In the present study, while it did not find a linear correlation between the DELTA-MELD and the number of portions of sugary sweets, the consumption of these foods was significantly lower in cirrhotic patients who subsequently improved compared with stable or worsened ones. This discrepancy could be due to the great heterogeneity of this food group in terms of fructose content. We also did not find an impact of fruit juice or soft drink consumption on DELTA-MELD. This result may have been influenced by the fact that only a minority of cirrhotic patients consumed these beverages.

Our results demonstrating the beneficial effect on the prognosis of cirrhotic patients of reducing the dietary intake of simple sugars, especially fructose, find support in data published in the field of NAFLD and MAFLD. Indeed, high fructose consumption exerts a negative metabolic effect in these patients on hepatic lipid synthesis, visceral adipose tissue accumulation, insulin resistance, increased uric acid and inflammation, and fibrosis progression [25,26,27,28,29]. Furthermore, it is known that patients with NAFLD and high amounts of visceral fat are at increased risk of insulin resistance [38]. This is in agreement with the stronger correlation between simple sugar consumption and changes in cirrhosis prognosis that we found in our study in patients with high visceral adiposity than in those with low visceral adiposity. Finally, in a recent study, fasting blood concentration of fructose was found to correlate positively with the prevalence of MAFLD, with liver fat, and with insulin resistance [39]. Our findings are also supported by data linking fructose and its metabolism to liver injury published in fields other than NAFLD or MAFLD. Indeed, in the field of alcoholic hepatitis, another LT indication in which the MELD score and its temporal changes have a predictive value [40,41,42], it has been reported that: (a) the activity of aldose reductase, the only enzyme that produces endogenous fructose, and the metabolites of this enzyme, fructose and uric acid, are higher in the livers of patients with this disease than in healthy livers; and (b) aldose reductase activity and serum uric acid concentration correlate positively with hepatocyte damage and the severity of alcoholic hepatitis [43]. Furthermore, the hepatic expression of aldose reductase is higher in patients with cirrhosis of different etiologies than in healthy control livers [44]. Finally, high serum uric acid levels have been found to correlate with serum transaminase values and with the incidence of hospitalizations and mortality of patients with cirrhosis [45].

Literature data concerning the role of carbohydrates in the diet of cirrhotic patients are scarce. For this reason, current dietary guidelines for cirrhotic patients do not include carbohydrate restrictions. The only exception is a generic reduction of carbohydrates, without mention of the type, in overweight/obese patients, as an indirect consequence of the recommendation to follow a low-calorie and protein-rich diet. Our study strongly suggests designing other studies to introduce the restriction of simple sugars into the guidelines. This is also necessary considering the increasing incidence of MAFLD as an etiology of cirrhosis in general and in the LT setting [19,20,22]. Our data are important not only for the management of patients with cirrhosis in general, but also for LT candidates. In fact, the temporal changes of the MELD score, when relevant as those of our study, can influence the possibility of joining the waiting list, prioritization in the waiting list, or delisting.

In our study, although the consumption of simple sugars by patients with liver cirrhosis did not differ from that of patients with chronic hepatitis or healthy controls, it was higher than that recommended for the general population. In fact, only 9% of our cirrhotic patients reported consuming simple sugars below 10% of total calories, as recommended by the WHO for the general population. The mean consumption of simple sugars of our cirrhotic patients was 16.2% of total calories, similar to the 16.4% reported in Greek cirrhotic patients [46] and lower than the 21.6% in French cirrhotic patients [47]. Thus, our data and the literature show that patients with cirrhosis consume excessive amounts of simple sugars. High consumption of fructose-containing foods such as jam and honey, which we have found to correlate with clinical worsening, has also been found in French cirrhotic patients.

Our data show that for a cirrhotic patient with an average weight of 80 kg, the median daily consumption of simple sugars associated with an improvement in MELD was 21.4 grams less per day compared with a cirrhotic patient who did not improve. This observation and our hypothesis that the mechanism of action is lower insulin resistance in improving patients are in agreement with a study demonstrating that even small amounts of dietary carbohydrate are sufficient to maintain hyperinsulinemia in these patients [48].

Although the nutritional data were collected only at the enrollment, we are confident that in the period between the MELD score evaluations the diet was not modified, given the precise indications provided to patients. We understand that factors other than food intake could affect the MELD score; however, we believe that our data are robust because we found strong linear correlations between food consumption and temporal changes in MELD, even after adjustment for cirrhosis etiologies.

Another result of our study, although obtained in a subgroup of patients in which a second CT scan was available 6 months after enrollment, is that a diet low in simple sugars is associated with a reduction in the VATI/SMI ratio over time. This is a further beneficial effect of the diet low in soluble sugars, as it is known that a reduction of skeletal muscle mass coupled with an increase in visceral fat are associated with an increased mortality of cirrhotic patient candidates for LT and with greater fibrosis in NALFD [49,50,51].

In conclusion, in our study we have, for the first time, investigated the effects of dietary carbohydrate consumption in cirrhotic patients on clinical outcomes. Our results show that, although cirrhotic patients consume similar amounts of simple sugars as healthy subjects and patients with chronic hepatitis, this consumption almost always exceeds the 10% of total calories suggested by the guidelines for the general population. Furthermore, our most important result is that, especially in cirrhotic patients with high visceral adipose tissue, the consumption of simple sugars and fructose-rich foods in the diet is independently associated with the worsening of the MELD score measured over 6 months. Finally, in a subgroup of patients with cirrhosis, we also found that the dietary consumption of simple sugars is greater in those who worsen their nutritional status over time with a relative depletion of muscle mass and increase in visceral fat. These results have clinical relevance for cirrhotic patients in general and for LT candidates and are a stimulus for designing further studies. If these studies confirm that a low consumption of simple sugars, and in particular of fructose, is beneficial for patients with cirrhosis and excess body fat, nutritional guidelines for these patients should be implemented.

## Figures and Tables

**Table 1:** Demographic, clinical, and nutritional characteristics of patients with cirrhosis, chronic hepatitis, and healthy controls.

	Cirrhotic Patients (*n* = 80)	Chronic Hepatitis Patients (*n* = 25)	Healthy Controls (*n* = 15)	*p*-Value Cirrhotic vs. Chronic Hepatitis Patients	*p*-Value Cirrhotic Patients vs. Healthy Controls
Age in years, median (IQR)	59.5 (53.3–63.0)	59.0 (52.6–70.8)	58.0 (51.0–63.0)	0.158	0.810
Gender, M (%)	70 (87.5)	17 (68.0)	12 (80)	0.024	0.426
BMI (kg/m^2^), median (IQR)	27.5 (24.6–31.3)	24.6(23.3–27.6)	24.9 (23.2–27.0)	0.006	0.017
Overweight/Obesity, *n* (%)	59 (73.8)	11 (44.0)	7 (46.7)	0.006	0.037
Diabetes, *n* (%)	21 (26.3)	0 (0)	0 (0)	<0.001	<0.001
Dyslipidemia, *n* (%)	13 (16.3)	0 (0)	0 (0)	<0.001	<0.001
Arterial hypertension, *n* (%)	19 (23.8)	0 (0)	0 (0)	<0.001	<0.001
Nutritional assessment:					
- SGA A, n (%)	73 (91.3)	25 (100)	15 (100)	0.491	0.650
- SGA B, n (%)	6 (7.5)	0 (0)	0 (0)		
- SGA C, n (%)	1 (1.3)	0 (0)	0 (0)		
MAC (cm), median (IQR)	30.0 (27.0–35.0)	29.3 (28.0–31.9)	32.0 (30.0–34.0)	0.582	0.417
Triceps skinfold measurements (mm), median (IQR)	13.6 (7.7–20.1)	13.6 (11.8–15.9)	9.9 (7.5–14.9)	0.972	0.111
Energy (Kcal/Kg/day), median (IQR)	20.8 (17.3–27.1)	23.5 (17.7–28.9)	25.8 (24.5–33.5)	0.232	0.005
Proteins dietary daily intake (g/Kg of body weight), median (IQR)	0.86 (0.66–1.11)	0.94 (0.78–1.12)	1.08 (0.86–1.41)	0.254	0.017
Fats dietary daily intake (g/Kg of body weight), median (IQR)	0.73 (0.56–1.06)	0.95 (0.66–1.14)	1.06 (0.81–1.29)	0.062	0.002
Carbohydrates dietary daily intake (g/Kg of body weight), median (IQR)	2.91 (2.24–3.68)	2.96 (2.33–3.53)	3.10 (2.64–3.80)	0.897	0.401
Starch dietary daily intake (g/Kg of body weight), median (IQR)	2.04 (1.60–2.45)	2.00 (1.57–2.28)	2.11 (1.54–2.70)	0.518	0.713
Simple sugars dietary daily intake (g/Kg of body weight), median (IQR)	0.84 (0.66–1.16)	0.92 (0.63–1.15)	0.99 (0.68–1.30)	0.712	0.307
Simple sugars dietary daily intake (% of total energy), median (IQR)	16.2 (13.5–21.2)	15.5 (12.1–20.9)	15.5 (10.6–20.4)	0.685	0.358
Vegetables (servings/day), median (IQR)	1.24 (0.71–1.90)	0.58 (0.32–1.08)	0.63 (0.42–1.10)	0.002	0.016
Fruit (servings/day), median (IQR)	1.56 (0.83–2.33)	1.49 (0.72–2.24)	0.47 (0.23–1.87)	0.741	0.011
Sum of added sugar, jam, and honey (servings/day), median (IQR)	0.67 (0.00–1.66)	0.67 (0.00–1.33)	0.33 (0.00–1.07)	0.988	0.813
Milk and yogurt (servings/day), median (IQR)	0.80 (0.00–1.50)	1.66 (0.55–2.17)	1.07 (0.33–2.00)	0.009	0.490
Soft drinks (servings/day), median (IQR)	0.00 (0.00–0.18)	0.00 (0.00–0.00)	0.00 (0.00–0.25)	0.011	0.861
Fruit juice (servings/day), median (IQR)	0.00 (0.00–0.33)	0.00 (0.00–0.00)	0.00 (0.00–0.33)	0.020	0.564
Sugary sweets (servings/day), median (IQR)	0.53 (0.11–1.00)	0.33 (0.00–1.07)	1.13 (0.33–1.47)	0.578	0.029

All data refer to baseline unless differently specified. Abbreviations: BMI, body mass index; IQR, interquartile range; MAC, mid-arm circumference; SGA, subjective global nutritional assessment.

**Table 2:** Demographic, clinical, and nutritional characteristics of patients with cirrhosis subgrouped according to their MELD score changes over time.

	Stable or Worsened MELD Score (*n* = 51)	Improved MELD Score (*n* = 29)	*p*-Value
Age in years, median (IQR)	60.0 (56.0–63.0)	59.0 (51.0–62.5)	0.170
Gender, M (%)	47 (92.2)	23 (79.3)	0.157
BMI, kg/m^2^, median (IQR)	27.6 (24.1–31.5)	27.4 (25.3–30.7)	0.964
Overweight/Obesity, *n* (%)	36 (70.6)	23 (79.3)	0.394
Diabetes, *n* (%)	14 (27.5)	7 (24.1)	0.746
Dyslipidemia, *n* (%)	9 (17.6)	4 (13.8)	0.760
Arterial hypertension, *n* (%)	16 (31.4)	3 (10.3)	0.054
Cirrhosis etiology:			
- Viral, n (%)	24 (47.1)	14 (48.3)	0.917
- Alcohol, n (%)	25 (49)	18 (62.1)	0.260
- MAFLD, n (%)	41 (80.4)	25 (86.2)	0.760
HCC, yes (%)	24 (47.1)	11 (37.9)	0.429
Ascites, *n* (%)	22 (43.1)	11 (37.9)	0.649
Serum AST, UI/L, median (IQR)	44.0 (32.0–77.0)	49.0 (33.5–69.0)	0.741
Serum ALT, UI/L, median (IQR)	34.0 (20.0–62.0)	31.0 (20.5–43.5)	0.814
MELD at baseline, median (IQR)	12.6 (10.0–14.8)	12.0 (11.0–14.9)	0.864
MELD after 6 months, median (IQR)	15.0 (12.0–19.0)	11.0 (9.0–13.0)	<0.001
Δ-MELD, median (IQR)	1.54 (0.39–3.19)	−0.80 (–2.36–−0.39)	<0.001
Charlson modified comorbidity index:			
- 0	20 (39.2)	17 (58.6)	0.229
- 1–2	25 (49)	9 (31)	
- >2	6 (11.8)	3 (10.3)	
Nutritional assessment:			
- SGA A, n (%)	47 (92.2)	26 (89.7)	0.585
- SGA B, n (%)	3 (5.9)	3 (10.3)	
- SGA C, n (%)	1 (2)	0 (0)	
MAC (cm), median (IQR)	30.0 (27.0–33.0)	31.0 (27.0–36.0)	0.868
Triceps skinfold measurements (mm), median (IQR)	13.8 (8.8–20.0)	11.8 (6.4–20.2)	0.386
VATI (cm^2^/m^2^), median (IQR)	38.1 (30.5–54.5)	41.1 (30.1–46.6)	0.572

All data refer to baseline unless differently specified. Abbreviations: AST, aspartate transaminase; ALT, alanine transaminase; BMI, body mass index; IQR, interquartile range; HCC, hepatocellular carcinoma; MAFLD, metabolic associated fatty liver disease; MAC, mid-arm circumference; MELD, model for end-stage liver disease; SGA, subjective global nutritional assessment; VATI, visceral adipose tissue index.

**Table 3:** Number of servings of foods containing simple sugars eaten per day by patients with cirrhosis according to changes in MELD score over time.

	Stable or Worsened MELD Score (*n* = 51)	Improved MELD Score (*n* = 29)	*p*-Values
Daily servings of the sum of added sugar, jam, and honey, median (IQR)	1.0 (0.00–2.0)	0.0(0.0–1.0)	0.002
Daily servings of fruit, median (IQR)	1.60 (1.0–2.55)	1.46 (0.60–1.80)	0.118
Daily servings of added sugar, jam, honey, and fruit, median (IQR)	2.91 (2.06–3.97)	1.66 (0.76–2.80)	0.002
Daily servings of sugary sweets, median (IQR)	0.67 (0.33–1.06)	0.27 (0.00–0.61)	0.005
Daily servings of added sugar, jam, honey, fruit, and sugary sweets, median (IQR)	3.66 (2.62–4.68)	2.33 (1.02–2.23)	<0.001
Daily servings of vegetables, median (IQR)	1.26 (0.93–1.90)	1.14 (0.33–1.95)	0.198
Daily servings of milk and yogurt, median (IQR)	0.80 (0.0–1.50)	1.16 (0.13–1.50)	0.373

All data refer to baseline. Servings have been defined according to the Italian Society of Human Nutrition (SINU) [36].

**Table 4:** Results of unadjusted and adjusted linear regression analyses of the association between changes in MELD score over time (DELTA-MELD) and selected daily dietary intakes in the entire cirrhotic study population.

Daily Dietary Intake	Unadjusted Analysis				Adjusted Analysis			
	B	95% Lower CI	95% Upper CI	*p*-Value	B	95% Lower CI	95% Upper CI	*p*-Value
Simple sugars (g/Kg of body weight)	1.544	0.327	2.761	0.014	1.602	0.390	2.814	0.010
Simple sugars (% of total energy)	0.148	0.064	0.233	0.001	0.158	0.074	0.242	0.0004
Sum of added sugar, jam, and honey (servings/Kg of body weight)	42.003	2.731	81.276	0.036	41.401	2.597	80.204	0.037
Fruit (servings/Kg of body weight)	36.806	−1.407	75.018	0.059	39.964	1.916	78.012	0.040
Sum of added sugar, jam, honey, and fruit (servings/Kg of body weight)	41.328	14.071	68.585	0.003	42.083	14.954	69.213	0.003
Sugary sweets (servings/Kg of body weight)	13.486	−49.766	76.737	0.672	4.829	-59.462	69.119	0.881

All multiple regression models were adjusted for age, gender, alcoholic etiology, viral etiology, and MAFLD etiology or, instead of MAFLD etiology, for VATI.

## Data Availability

Data are available upon request.

## Supplementary Materials

The following supporting information can be downloaded at https://www.mdpi.com/article/10.3390/nu15071575/s1, Table S1: Clinical, demographic, and nutritional characteristics of cirrhotic patients with high or low visceral fat according to their MELD score changes over time; Table S2: Results of unadjusted and adjusted linear regression analyzes of the association between changes in MELD score over time (DELTA-MELD) and selected daily dietary intakes in cirrhotic patients with high or low visceral adiposity. Figure S1: Boxplots of the number of servings of some simple sugar-containing food categories consumed each day per unit of body weight, based on temporal changes in MELD score by all cirrhotic patients. Figure S2: Boxplots of daily dietary intake of simple sugars expressed per unit of body weight, based on temporal changes in visceral adipose tissue, skeletal muscle mass, and their ratio.
