# Supplementary material for: A Low Daily Intake of Simple Sugars in the Diet Is Associated with Improved Liver Function in Cirrhotic Liver Transplant Candidates
Source: Nutrients. 2023 Mar 24;15(7):1575. doi: 10.3390/nu15071575 (PMC10097197; doi:10.3390/nu15071575)
Supplement: Supplementary file 1 [file nutrients-15-01575-s001.zip › Supplementary Figure S1.pdf]

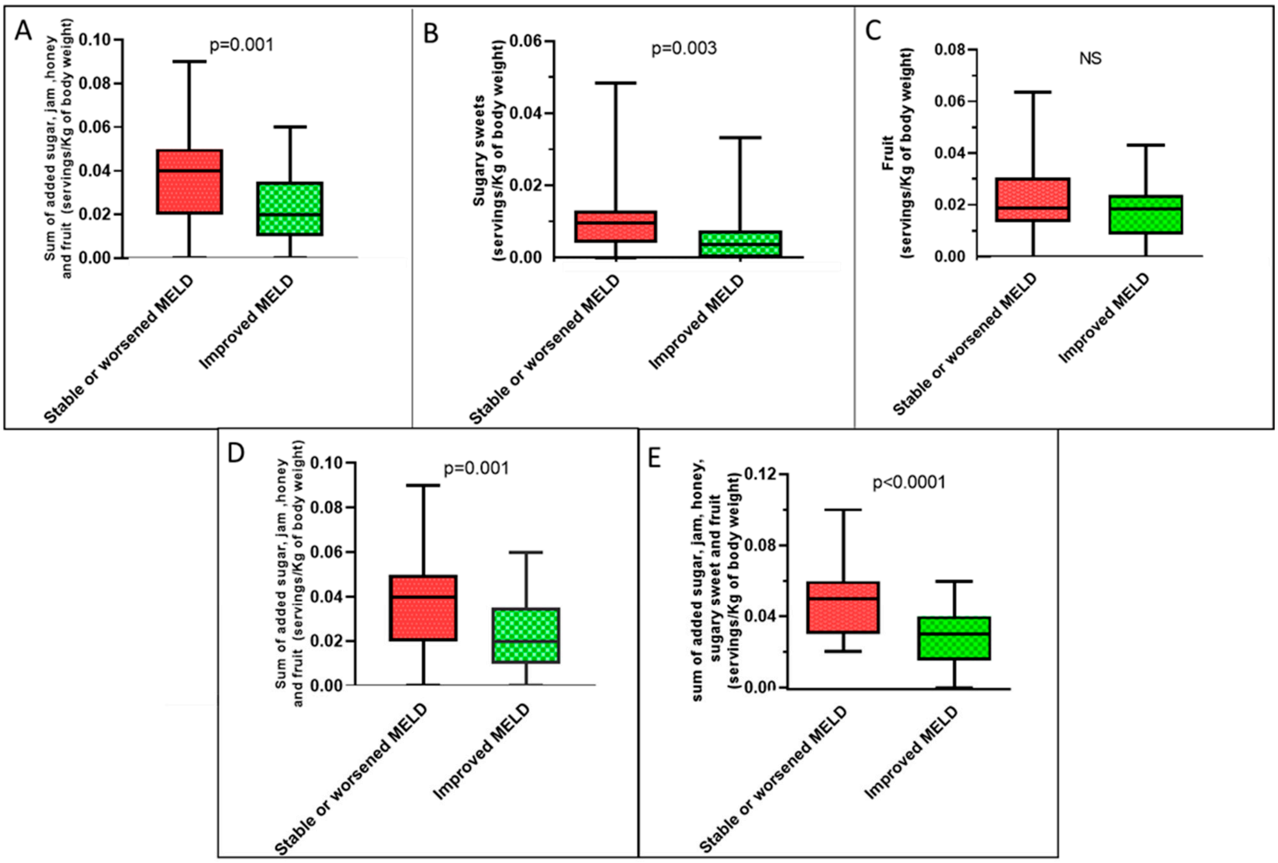

Supplementary Figure S1. Boxplots of the number of servings of some simple sugar-containing food categories consumed each day per unit of body weight, based on temporal changes in MELD score by all cirrhotic patients. (A) servings of the sum of added sugars, jam and honey; (B) servings of sugary sweets; (C) servings of fruit; (D) servings of the sum of added sugars, jam, honey and fruit; (E) servings of the sum of added sugars, jam, honey, sugary sweets and fruit.
