# Supplementary material for: A Low Daily Intake of Simple Sugars in the Diet Is Associated with Improved Liver Function in Cirrhotic Liver Transplant Candidates
Source: Nutrients. 2023 Mar 24;15(7):1575. doi: 10.3390/nu15071575 (PMC10097197; doi:10.3390/nu15071575)
Supplement: Supplementary file 1 [file nutrients-15-01575-s001.zip › Supplementary Figure S2.pdf]

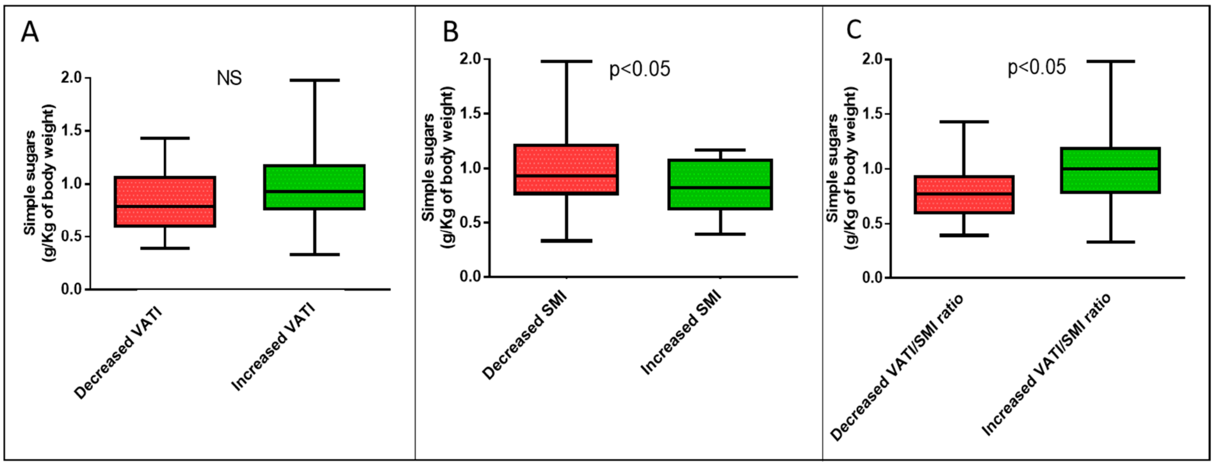

Supplementary Figure S2. Boxplots of daily dietary intake of simple sugars expressed per unit of body weight, based on temporal changes in visceral adipose tissue, skeletal muscle mass, and their ratio. (A) VATI; (B) SMI; (C) VATI/SMI ratio.
