# Supplementary material for: A Low Daily Intake of Simple Sugars in the Diet Is Associated with Improved Liver Function in Cirrhotic Liver Transplant Candidates
Source: Nutrients. 2023 Mar 24;15(7):1575. doi: 10.3390/nu15071575 (PMC10097197; doi:10.3390/nu15071575)
Supplement: Supplementary file 1 [file nutrients-15-01575-s001.zip › Supplementary Table S1.pdf]

Supplementary Table S1. Clinical, demographic and nutritional characteristics of cirrhotic patients with high or low visceral fat according to their MELD score changes overtime.

	High visceral fat				Low visceral fat		
	Stable or worsened MELD score (n=25)	Improved MELD score (n=15)	P value		Stable or worsened MELD score (n=26)	Improved MELD score (n=14)	P value
Age, years, median (IQR)	60.0 (58.0-62.0)	59.0 (51.0-63.0)	0.059		58.5 (51.8-64.0)	57.0 (51.8-62.3)	0.620
Gender, M (%)	23 (92)	12 (80)	0.345		24 (92.3)	11 (78.6)	0.322
BMI, median (IQR)	29.6 (26.7-32.5)	28.2 (26.2-33.1)	0.472		25.8 (23.4-28.7)	25.5 (23.0-30.3)	0.877
Overweight/Obesity, n (%)	20 (80)	14 (93.3)	0.381		16 (61.5)	9 (64.3)	1.000
Diabetes, n (%)	7 (28)	4 (26.7)	1.000		7 (26.9)	3 (21.4)	1.000
Dyslipidaemia, n (%)	4 (16)	2 (13.3)	1.000		5 (19.2)	2 (14.3)	1.000
Arterial hypertension, n (%)	9 (36)	3 (20)	0.477		7 (26.9)	14 (100)	0.075
Cirrhosis aetiology:<br><br>Viral, n (%)<br>Alcohol, n (%)<br>MAFLD, n (%)	 <br>8 (32)<br>18 (72)<br>22 (88)	 <br>8 (53.3)<br>11 (73.3)<br>15 (100)	 <br>0.182<br>1.000<br>0.279		 <br>16 (61.5)<br>7 (26.9)<br>19 (73.1)	 <br>6 (42.9)<br>7 (50)<br>10 (71.4)	 <br>0.257<br>0.144<br>1.000
Ascites, n (%)	14 (56)	8 (53.3)	0.870		1 5(57.7)	10 (71.4)	0.502
HCC, yes (%)	13 (52)	6 (40)	0.462		11 (42.3)	5 (35.7)	0.746
Serum AST, UI/L, median (IQR)	37.0 (28.0-54.5)	47.0 (37.0-77.0)	0.133		58.8 (35.8-91.0)	51.0 (31.5-68.0)	0.332
Serum ALT, UI/L, median (IQR)	25.0 (18.0-38.0)	27.0 (20.0-50.0)	0.472		42.5 (24.0-84.8)	33.5 (23.5-43.3)	0.254
MELD at baseline, median (IQR)	12.9 (9.4-15.1)	11.7 (9.5-14.3)	0.507		12.0 (10.2-14.9)	12.7 (11.2-15.3)	0.440
MELD after 6 months, median (IQR)	15.0 (12.5-17.0)	11.0 (9.0-13.0)	**0.001**		15.0 (11.9-19.0)	11.0 (8.8-14.3)	**0.023**
Δ-MELD, median (IQR)	1.47 (0.33-2.86)	-0.97 (-2.31- -0.42)	**<0.001**		1.67 (0.37-3.79)	-0.65 (-2.48- -0.35)	**<0.001**
Charlson Modified comorbidity index:<br>0<br>1-2<br>>2	 11 (44)<br>11 (44)<br>3 (12)	 8 (53.3)<br>5 (33.3)<br>2 (13.3)	0.798		 9 (34.6)<br>14 (53.8)<br>3 (11.5)	 9 (64.3)<br>4 (28.6)<br>1 (7.1)	0.197
Nutritional assessment:<br>- SGA A, n (%)<br>-SGA B, n (%)<br>-SGA C, n (%)	 23 (92)<br>1 (4)<br>1 (4)	 14 (93.3)<br>1 (6.7)<br>0 (0)	0.692		 24 (92.3)<br>2 (7.7)<br>0 (0)	 12 (85.7)<br>2 (14.3)<br>0 (0)	0.602
MAC, (cm), median (IQR)	30.0 (26.5-34.5)	33.0 (28.0-37.0)	0.181		30.0 (27.8-33.0)	27.5 (24.0-33.8)	0.159
Triceps skinfold measurements (mm), median (IQR)	16.8 (9.4-20.3)	13.7 (9.3-23.4)	0.934		12.0 (6.8-19.1)	8.3 (5.5-17.1)	0.097
VATI (cm^2/m^2), median (IQR)	54.5 (48.5-64.3)	45.7 (42.6-54.2)	**0.035**		30.8 (23.7-35.8)	30.1 (22.9-36.4)	0.824

Abbreviations: all data refer at baseline unless differently specified
Abbreviations: AST, aspartate transaminase; ALT alanine transaminase; BMI, body mass index; IQR, interquartile range; HCC, hepatocellular carcinoma; MAFLD, metabolic associated fatty liver disease; MAC, mid-arm circumference; MELD, model for end stage liver disease; SGA subjective global nutritional assessment; VATI, visceral adipose tissue index.
