# Supplementary material for: A Low Daily Intake of Simple Sugars in the Diet Is Associated with Improved Liver Function in Cirrhotic Liver Transplant Candidates
Source: Nutrients. 2023 Mar 24;15(7):1575. doi: 10.3390/nu15071575 (PMC10097197; doi:10.3390/nu15071575)
Supplement: Supplementary file 1 [file nutrients-15-01575-s001.zip › Supplementary Table S2.pdf]

Table S2. Results of unadjusted and adjusted linear regression analyzes of the association between changes in MELD score over time (DELTA MELD) and selected daily dietary intakes in cirrhotic patients with high or low visceral adiposity.

	**Daily dietary intake**	**Unadjusted analysis**					**Adjusted analysis**			
		**B**	**95% lower CI**	**95% upper CI**	**P value**		**B**	**95% lower CI**	**95% upper CI**	**P value**
**HIGH VATI**	**Simple sugars (g/Kg of body weight)**	**2.085**	**0.470**	**3.647**	**0.012**		**2.285**	**0.617**	**3.954**	**0.009**
	**Simple sugars (% of total energy)**	**0.152**	**0.043**	**0.260**	**0.007**		**0.169**	**0.055**	**0.283**	**0.005**
	**Sum of added sugar, jam and honey (servings/Kg of body weight)**	**67.574**	**6.853**	**128.296**	**0.030**		**64.741**	**3.302**	**126.179**	**0.039**
	**Fruit (servings/Kg of body weight)**	35.910	-16.755	88.575	0.176		32.001	-21.922	85.924	0.237
	**Sum of added sugar, jam, honey and fruit (servings/Kg of body weight)**	**45.862**	**8.672**	**83.044**	**0.017**		**48.970**	**10.436**	**87.503**	**0.014**
	**Sugary sweet (servings/Kg of body weight)**	2.405	-78.212	83.022	0.952		-4.390	-94.033	85.253	0.921
**LOW VATI**	**Simple sugars (g/Kg of body weight)**	0.985	-0.957	2.927	0.311		0.406	-1.572	2.384	0.680
	**Simple sugars (% of total energy)**	**0.157**	**0.015**	**0.299**	**0.032**		**0.161**	**0.020**	**0.302**	**0.026**
	**Sum of added sugar, jam and honey (servings/Kg of body weight)**	26.221	-27.747	80.189	0.332		23.103	-30.535	76.740	0.388
	**Fruit (servings/Kg of body weight)**	38.741	-19.855	97.337	0.189		25.901	-42.689	94.491	0.448
	**Sum of added sugar, jam, honey and fruit (servings/Kg of body weight)**	37.488	-4.530	79.505	0.079		27.216	-17.093	71.524	0.221
	**Sugary sweet (servings/Kg of body weight)**	33.910	-73.864	141.684	0.528		48.107	-57.902	154.116	0.363
							All multiple regression models were adjusted for age, gender, alcoholic aetiology and viral aetiology. B is the unstandardized coefficient of multiple linear regression.			
